# Supplementary material for: Assessing the COVID-19 legacy on hand hygiene: Retrospective observational before–after study of compliance and alcohol-based
Source: PLOS Glob Public Health. 2026 Feb 27;6(2):e0005210. doi: 10.1371/journal.pgph.0005210 (PMC12948101; doi:10.1371/journal.pgph.0005210)
Supplement: S1 Table — The table presents direct observational data collected in adult, pediatric, and neonatal intensive care units, stratified by date, hospital sector, and professional category. Hand hygiene opportunities were classified according to the WHO Five Moments: (1) before contact with the patient; (2) before performing an aseptic procedure; (3) after risk of exposure to bodily fluids; (4) after contact with the patient; and (5) after contact with surfaces near the patient. Abbreviations: S, hand hygiene performed; N, hand hygiene not performed; A, alcohol-based hand rub used; 0, no opportunity observed. (DOCX) [file pgph.0005210.s001.docx]

**Supplementary DataSet**

**S1 Table.** Opportunities for Hand Hygiene According to Professional Category and WHO’s Five Moments for Hand Hygiene During the COVID-19 Pandemic. N: not hand hygiene compliance; A: alcohol; S: soap; 0: not Applicable.

| **Date** | **Sector** | **Professional Category** | **Moment 1: Before contact with the patient** | **Moment 2: Before performing the aseptic procedure** | **Moment 3: After risk of exposure to bodily fluids** | **Moment 4: After contact with the patient** | **Moment 5: After contact with surfaces near the patient** |
| --- | --- | --- | --- | --- | --- | --- | --- |
| 10/29/2021 | Adult ICU | Nurse | N | 0 | 0 | 0 | S |
| 10/29/2021 | Pediatric ICU | Nurse | N | 0 | 0 | S | 0 |
| 10/29/2021 | Adult ICU | Physiotherapist | S | 0 | 0 | S | 0 |
| 11/03/2021 | Adult ICU | Nurse | S | 0 | 0 | S | 0 |
| 11/03/2021 | Adult ICU | Doctor | N | 0 | 0 | N | 0 |
| 11/03/2021 | Adult ICU | Nurse | 0 | 0 | 0 | 0 | A |
| 11/03/2021 | Adult ICU | Doctor | N | N | A | A | 0 |
| 11/03/2021 | Adult ICU | Laboratory technician | 0 | 0 | N | N | 0 |
| 11/03/2021 | Adult ICU | Nursing Technicians | N | 0 | 0 | S | 0 |
| 11/03/2021 | Adult ICU | Doctor | N | N | A | A | 0 |
| 11/03/2021 | Adult ICU | Nurse | A | 0 | 0 | S | N |
| 11/03/2021 | Adult ICU | Nursing student | A | 0 | 0 | S | N |
| 11/03/2021 | Adult ICU | Medical student | N | 0 | 0 | S | 0 |
| 11/03/2021 | Adult ICU | Medical student | 0 | 0 | S | S | 0 |
| 11/03/2021 | Pediatric ICU | Nurse | N | 0 | 0 | A | 0 |
| 11/03/2021 | Adult ICU | Nurse | A | 0 | 0 | S | N |
| 11/03/2021 | Adult ICU | Nurse | A | A | 0 | S | N |
| 11/03/2021 | Adult ICU | Nurse | N | 0 | 0 | S | 0 |
| 11/03/2021 | Pediatric ICU | Nursing Technicians | 0 | 0 | 0 | 0 | N |
| 11/03/2021 | Pediatric ICU | Nurse | N | N | 0 | S | S |
| 11/03/2021 | Adult ICU | Nurse | N | 0 | N | S | S |
| 11/03/2021 | Neonatal ICU | Nurse | N | 0 | 0 | N | N |
| *Continuation S1 Table:* | | | | | | | |
| 11/03/2021 | Neonatal ICU | Nurse | A | 0 | 0 | N | N |
| 11/03/2021 | Pediatric ICU | Doctor | A | 0 | 0 | S | S |
| 11/03/2021 | Adult ICU | Nurse | N | 0 | 0 | S | S |
| 11/03/2021 | Adult ICU | Nurse | S | N | S | S | N |
| 11/03/2021 | Adult ICU | Nurse | N | 0 | 0 | 0 | N |
| 11/03/2021 | Adult ICU | Nurse | A | 0 | 0 | S | 0 |
| 11/03/2021 | Pediatric ICU | Nurse | S | 0 | 0 | 0 | S |
| 11/03/2021 | Adult ICU | Nursing Technicians | S | 0 | 0 | S | 0 |
| 11/03/2021 | Pediatric ICU | Nurse | N | 0 | 0 | 0 | A |
| 11/03/2021 | Pediatric ICU | Medical student | N | 0 | 0 | A | 0 |
| 11/03/2021 | Adult ICU | Doctor | N | 0 | 0 | A | 0 |
| 11/03/2021 | Pediatric ICU | Nurse | A | 0 | 0 | N | 0 |
| 11/03/2021 | Adult ICU | Nurse | A | 0 | S | S | S |
| 11/03/2021 | Adult ICU | Doctor | A | 0 | 0 | S | 0 |
| 11/03/2021 | Adult ICU | Nursing Technicians | N | 0 | 0 | S | S |
| 11/03/2021 | Pediatric ICU | Nurse | N | 0 | 0 | S | 0 |
| 11/03/2021 | Adult ICU | Nurse | N | 0 | 0 | S | 0 |
| 11/03/2021 | Pediatric ICU | Nurse | 0 | 0 | N | N | N |
| 11/03/2021 | Adult ICU | Physiotherapist | N | 0 | S | S | S |
| 11/03/2021 | Adult ICU | Doctor | N | N | A | A | A |
| 11/03/2021 | Adult ICU | Physiotherapist | A | A | A | A | 0 |
| 11/03/2021 | Adult ICU | Nurse | N | N | S | S | 0 |
| 11/03/2021 | Adult ICU | Nursing Technicians | N | N | 0 | S | 0 |
| 11/03/2021 | Adult ICU | Nursing Technicians | N | N | 0 | S | 0 |
| 11/03/2021 | Adult ICU | Nursing Technicians | N | 0 | S | S | S |
| 11/03/2021 | Neonatal ICU | Physiotherapist | S | S | N | S | N |
| 11/03/2021 | Adult ICU | Physiotherapist | A | A | N | S | N |
| 11/03/2021 | Adult ICU | Nursing Technicians | 0 | 0 | S | S | S |
| 11/03/2021 | Adult ICU | Medical student | A | 0 | 0 | S | S |
| 11/03/2021 | Adult ICU | Nurse | N | N | S | S | 0 |
| 11/03/2021 | Neonatal ICU | Doctor | A | 0 | 0 | S | 0 |
| 11/03/2021 | Pediatric ICU | Nurse | N | 0 | 0 | S | N |
| *Continuation S1 Table:* | | | | | | | |
| 11/03/2021 | Adult ICU | Doctor | N | 0 | 0 | N | N |
| 11/03/2021 | Pediatric ICU | Nursing Technicians | S | 0 | 0 | S | 0 |
| 11/03/2021 | Adult ICU | Nurse | N | N | S | S | 0 |
| 11/04/2021 | Adult ICU | Nurse | A | 0 | 0 | A | A |
| 11/04/2021 | Adult ICU | Nurse | A | 0 | 0 | S | 0 |
| 11/04/2021 | Adult ICU | Nursing Technicians | A | 0 | N | 0 | N |
| 11/04/2021 | Adult ICU | Doctor enfermagem | S | 0 | 0 | S | N |
| 11/04/2021 | Adult ICU | Nurse | S | 0 | 0 | S | N |
| 11/04/2021 | Adult ICU | Doctor | S | S | 0 | S | 0 |
| 11/04/2021 | Adult ICU | Nursing student | S | A | 0 | S | N |
| 11/04/2021 | Adult ICU | Doctor | S | 0 | 0 | A | N |
| 11/04/2021 | Adult ICU | Nurse | N | 0 | 0 | S | S |
| 11/04/2021 | Adult ICU | Doctor | N | 0 | 0 | S | 0 |
| 11/05/2021 | Neonatal ICU | Nurse | S | 0 | 0 | S | 0 |
| 11/05/2021 | Neonatal ICU | Nurse | A | 0 | 0 | A | 0 |
| 11/05/2021 | Neonatal ICU | Physiotherapist | S | 0 | 0 | S | 0 |
| 11/05/2021 | Neonatal ICU | Nurse | S | 0 | 0 | S | N |
| 11/05/2021 | Neonatal ICU | Nurse | S | 0 | 0 | S | N |
| 11/05/2021 | Adult ICU | Medical student | S | S | S | S | S |
| 11/07/2021 | Adult ICU | Nursing Technicians | N | 0 | 0 | A | 0 |
| 11/07/2021 | Adult ICU | Physiotherapist | S | 0 | 0 | S | 0 |
| 11/07/2021 | Adult ICU | Nursing Technicians | N | 0 | 0 | S | 0 |
| 11/07/2021 | Adult ICU | Nursing Technicians | N | 0 | 0 | N | 0 |
| 11/09/2021 | Pediatric ICU | Nurse | S | 0 | 0 | S | S |
| 11/09/2021 | Adult ICU | Nurse | N | 0 | 0 | S | S |
| 11/09/2021 | Adult ICU | Nurse | N | 0 | 0 | S | S |
| 11/09/2021 | Pediatric ICU | Nurse | A | 0 | 0 | S | S |
| 11/11/2021 | Adult ICU | Nursing student | A | 0 | N | S | N |
| 11/11/2021 | Adult ICU | Nurse | A | 0 | 0 | S | N |
| 11/11/2021 | Adult ICU | Medical student | A | 0 | 0 | S | 0 |
| 11/11/2021 | Adult ICU | Nurse | N | 0 | 0 | N | 0 |
| 11/12/2021 | Pediatric ICU | Medical student | N | 0 | 0 | S | S |
| *Continuation S1 Table:* | | | | | | | |
| 11/12/2021 | Neonatal ICU | Doctor | N | 0 | 0 | A | A |
| 11/12/2021 | Pediatric ICU | Medical student | S | 0 | 0 | S | S |
| 11/12/2021 | Pediatric ICU | Physiotherapist | S | S | S | S | S |
| 11/14/2021 | Adult ICU | Nurse | N | N | S | S | 0 |
| 11/14/2021 | Adult ICU | Nurse | N | 0 | 0 | S | 0 |
| 11/18/2021 | Adult ICU | Nurse | S | 0 | 0 | S | S |
| 11/18/2021 | Adult ICU | Doctor | A | 0 | 0 | N | 0 |
| 11/18/2021 | Neonatal ICU | Doctor | A | 0 | S | S | 0 |
| 11/18/2021 | Neonatal ICU | Nurse | A | 0 | 0 | S | 0 |
| 11/18/2021 | Neonatal ICU | Nursing student | S | 0 | 0 | N | 0 |
| 11/18/2021 | Adult ICU | Nursing Technicians | N | 0 | 0 | S | S |
| 11/18/2021 | Pediatric ICU | Nurse | A | 0 | S | S | 0 |
| 11/18/2021 | Adult ICU | Nurse | N | 0 | 0 | N | N |
| 11/18/2021 | Pediatric ICU | Nurse | A | 0 | 0 | S | N |
| 11/18/2021 | Adult ICU | Nurse | N | N | S | S | 0 |
| 11/18/2021 | Adult ICU | Nursing Technicians | N | 0 | S | 0 | S |
| 11/25/2021 | Adult ICU | Doctor | A | 0 | 0 | S | 0 |
| 11/25/2021 | Adult ICU | Nurse | A | 0 | N | S | N |
| 11/25/2021 | Adult ICU | Doctor | S | 0 | 0 | S | 0 |
| 11/25/2021 | Pediatric ICU | Nurse | A | 0 | 0 | S | N |
| 11/25/2021 | Pediatric ICU | Nurse | S | 0 | 0 | N | 0 |
| 11/25/2021 | Neonatal ICU | Pharmaceutical | S | 0 | 0 | S | 0 |
| 11/25/2021 | Neonatal ICU | Doctor | A | 0 | 0 | A | 0 |
| 11/25/2021 | Neonatal ICU | Nurse | A | 0 | N | S | N |
| 11/25/2021 | Adult ICU | Nurse | S | S | S | S | 0 |
| 11/25/2021 | Adult ICU | Nursing Technicians | N | 0 | S | S | S |
| 11/29/2021 | Pediatric ICU | Nurse | N | 0 | 0 | S | 0 |
| 11/29/2021 | Adult ICU | Nurse | N | 0 | S | S | 0 |
| 11/30/2021 | Pediatric ICU | Nurse | N | 0 | 0 | A | N |
| 12/02/2021 | Neonatal ICU | Nursing Technicians | S | 0 | 0 | S | 0 |
| 12/02/2021 | Neonatal ICU | Nurse | S | 0 | 0 | S | 0 |
| 12/02/2021 | Neonatal ICU | Physiotherapist | A | 0 | S | S | N |
| *Continuation S1 Table:* | | | | | | | |
| 12/02/2021 | Neonatal ICU | Nurse | A | 0 | S | S | N |
| 12/02/2021 | Neonatal ICU | Doctor | S | 0 | 0 | N | 0 |
| 12/02/2021 | Neonatal ICU | Doctor | A | 0 | 0 | S | 0 |
| 12/02/2021 | Adult ICU | Nurse | A | 0 | 0 | S | N |
| 12/02/2021 | Adult ICU | Nurse | S | 0 | 0 | S | 0 |
| 12/02/2021 | Pediatric ICU | Nurse | A | 0 | N | N | N |
| 12/02/2021 | Pediatric ICU | Nurse | S | 0 | 0 | S | 0 |
| 12/02/2021 | Adult ICU | Nurse | N | N | 0 | S | S |
| 12/02/2021 | Adult ICU | Doctor | S | S | 0 | S | 0 |
| 12/02/2021 | Adult ICU | Nurse | S | S | 0 | S | 0 |
| 12/07/2021 | Adult ICU | Nurse | A | N | N | A | N |
| 12/07/2021 | Neonatal ICU | Nurse | A | N | N | A | N |
| 12/08/2021 | Adult ICU | Nursing Technicians | N | 0 | 0 | 0 | N |
| 12/08/2021 | Adult ICU | Nursing Technicians | N | 0 | 0 | 0 | A |
| 12/10/2021 | Neonatal ICU | Doctor | S | 0 | 0 | S | 0 |
| 12/10/2021 | Neonatal ICU | Physiotherapist | A | 0 | 0 | S | N |
| 12/10/2021 | Neonatal ICU | Nurse | S | 0 | 0 | S | 0 |
| 12/10/2021 | Neonatal ICU | Nurse | A | 0 | N | S | N |
| 12/10/2021 | Adult ICU | Nurse | A | 0 | 0 | S | N |
| 12/10/2021 | Adult ICU | Nursing Technicians | S | 0 | 0 | A | 0 |
| 12/10/2021 | Adult ICU | Physiotherapist | A | 0 | N | S | N |
| 12/10/2021 | Adult ICU | Nurse | N | 0 | 0 | N | 0 |
| 12/10/2021 | Adult ICU | Nursing Technicians | N | 0 | 0 | A | A |
| 12/16/2021 | Neonatal ICU | Nurse | S | 0 | 0 | N | N |
| 12/16/2021 | Neonatal ICU | Nurse | N | N | N | N | N |
| 12/16/2021 | Neonatal ICU | Nurse | S | 0 | 0 | A | 0 |
| 12/16/2021 | Adult ICU | Nurse | N | N | N | N | N |
| 12/16/2021 | Adult ICU | Nurse | A | 0 | 0 | S | 0 |
| 12/16/2021 | Adult ICU | Nurse | A | 0 | 0 | S | 0 |
| 12/16/2021 | Neonatal ICU | Nursing Technicians | N | 0 | 0 | A | A |
| 12/17/2021 | Neonatal ICU | Physiotherapist | A | 0 | 0 | S | 0 |
| 12/17/2021 | Neonatal ICU | Nurse | S | 0 | 0 | S | 0 |
| *Continuation S1 Table:* | | | | | | | |
| 12/17/2021 | Neonatal ICU | Nurse | N | 0 | 0 | S | N |
| 12/17/2021 | Neonatal ICU | Doctor | S | 0 | 0 | S | 0 |
| 12/17/2021 | Adult ICU | Physiotherapist | A | 0 | 0 | S | 0 |
| 12/17/2021 | Adult ICU | Nurse | S | 0 | 0 | S | 0 |
| 12/17/2021 | Adult ICU | Nursing Technicians | S | 0 | 0 | S | 0 |
| 12/20/2021 | Adult ICU | Nurse | A | A | 0 | A | 0 |
| 12/20/2021 | Pediatric ICU | Doctor | N | N | 0 | N | 0 |
| 12/20/2021 | Neonatal ICU | Nurse | A | A | 0 | A | 0 |
| 12/27/2021 | Adult ICU | Nurse | N | 0 | 0 | 0 | N |
| 12/29/2021 | Adult ICU | Nurse | N | 0 | 0 | 0 | S |
| 12/29/2021 | Adult ICU | Doctor | S | S | S | S | 0 |
| 01/18/2022 | Pediatric ICU | Nurse | N | N | 0 | S | N |
| 01/18/2022 | Pediatric ICU | Nursing student | N | N | 0 | N | N |
| 01/24/2022 | Adult ICU | Nurse | N | 0 | S | S | 0 |
| 01/28/2022 | Adult ICU | Nurse | A | N | 0 | A | 0 |
| 02/01/2022 | Adult ICU | Nurse | N | 0 | S | S | N |
| 02/01/2022 | Neonatal ICU | Doctor | A | 0 | 0 | S | S |
| 02/01/2022 | Pediatric ICU | Nurse | S | S | S | S | N |
| 02/01/2022 | Adult ICU | Nurse | A | 0 | S | S | 0 |
| 02/01/2022 | Pediatric ICU | Nursing student | S | 0 | 0 | S | 0 |
| 03/03/2022 | Neonatal ICU | Physiotherapist | A | 0 | 0 | 0 | S |
| 03/03/2022 | Adult ICU | Nurse | A | 0 | 0 | 0 | S |
| 03/03/2022 | Adult ICU | Nurse | S | 0 | 0 | S | 0 |
| 03/03/2022 | Adult ICU | Nurse | A | 0 | N | S | N |
| 03/07/2022 | Neonatal ICU | Nurse | A | 0 | 0 | A | 0 |
| 03/07/2022 | Neonatal ICU | Nurse | A | 0 | 0 | A | 0 |
| 03/08/2022 | Adult ICU | Nurse | A | 0 | 0 | A | N |
| 03/10/2022 | Neonatal ICU | Nurse | S | S | S | S | 0 |
| 03/10/2022 | Neonatal ICU | Doctor | N | 0 | 0 | S | N |
| 03/10/2022 | Neonatal ICU | Nurse | S | 0 | 0 | S | S |
| 03/10/2022 | Neonatal ICU | Doctor | 0 | 0 | 0 | A | 0 |
| 03/10/2022 | Neonatal ICU | Nurse | S | 0 | S | S | 0 |
| *Continuation S1 Table:* | | | | | | | |
| 03/10/2022 | Neonatal ICU | Doctor | N | 0 | 0 | 0 | N |
| 03/10/2022 | Adult ICU | Nurse | 0 | 0 | 0 | 0 | S |
| 03/10/2022 | Pediatric ICU | Doctor | 0 | 0 | 0 | A | A |
| 03/10/2022 | Pediatric ICU | Physiotherapist | S | 0 | 0 | S | N |
| 03/22/2022 | Adult ICU | Nurse | S | 0 | 0 | 0 | A |
| 03/22/2022 | Adult ICU | Nursing Technicians | A | 0 | 0 | A | 0 |
| 03/22/2022 | Adult ICU | Nursing Technicians | N | 0 | 0 | 0 | S |
| 03/22/2022 | Adult ICU | Nursing Technicians | N | 0 | 0 | 0 | S |
| 03/22/2022 | Neonatal ICU | Nurse | S | 0 | 0 | 0 | S |
| 03/22/2022 | Neonatal ICU | Nurse | S | 0 | 0 | S | 0 |
| 03/28/2022 | Adult ICU | Nurse | A | A | 0 | N | N |
| 03/28/2022 | Adult ICU | Nurse | N | 0 | 0 | N | N |
| 03/28/2022 | Adult ICU | Nurse | A | A | 0 | S | S |
| 03/28/2022 | Neonatal ICU | Doctor | N | 0 | 0 | S | S |
| 03/31/2022 | Neonatal ICU | Nurse | A | 0 | 0 | S | 0 |
| 03/31/2022 | Neonatal ICU | Physiotherapist | A | 0 | 0 | A | 0 |
| 03/31/2022 | Neonatal ICU | Nurse | 0 | 0 | A | A | A |
| 03/31/2022 | Neonatal ICU | Nurse | 0 | 0 | S | S | S |
| 03/31/2022 | Neonatal ICU | Nurse | S | S | S | S | 0 |
| 03/31/2022 | Neonatal ICU | Nurse | A | 0 | 0 | A | 0 |
| 03/31/2022 | Neonatal ICU | Physiotherapist | 0 | 0 | 0 | S | S |
| 03/31/2022 | Neonatal ICU | Nurse | N | 0 | 0 | S | 0 |
| 03/31/2022 | Neonatal ICU | Nurse | S | 0 | S | S | 0 |
| 03/31/2022 | Adult ICU | Doctor | N | 0 | 0 | S | 0 |
| 03/31/2022 | Adult ICU | Nurse | A | 0 | 0 | S | 0 |
| 03/31/2022 | Adult ICU | Doctor | A | 0 | S | S | 0 |
| 03/31/2022 | Adult ICU | Doctor | S | 0 | 0 | S | 0 |
| 03/31/2022 | Adult ICU | Doctor | 0 | 0 | S | S | S |
| 03/31/2022 | Pediatric ICU | Nurse | A | 0 | 0 | S | 0 |
| 03/31/2022 | Pediatric ICU | Physiotherapist | S | S | S | S | S |
| 03/31/2022 | Pediatric ICU | Doctor | A | 0 | 0 | 0 | N |
| 04/03/2022 | Pediatric ICU | Doctor | N | 0 | 0 | 0 | N |
| *Continuation S1 Table:* | | | | | | | |
| 04/03/2022 | Adult ICU | Nurse | S | S | S | S | S |
| 04/05/2022 | Adult ICU | Nurse | N | A | S | S | N |
| 04/14/2022 | Neonatal ICU | Nurse | 0 | 0 | S | S | S |
| 04/14/2022 | Neonatal ICU | Physiotherapist | S | S | S | S | S |
| 04/14/2022 | Neonatal ICU | Nurse | S | S | S | S | S |
| 04/14/2022 | Neonatal ICU | Nurse | 0 | 0 | S | S | 0 |
| 04/14/2022 | Neonatal ICU | Doctor | S | S | S | S | 0 |
| 04/14/2022 | Pediatric ICU | Nurse | S | S | S | S | 0 |
| 04/14/2022 | Adult ICU | Nurse | 0 | 0 | 0 | S | S |
| 04/14/2022 | Adult ICU | Nurse | S | S | S | S | S |
| 04/14/2022 | Pediatric ICU | Physiotherapist | S | S | S | S | 0 |
| 04/14/2022 | Adult ICU | Nurse | 0 | 0 | S | S | S |
| 04/14/2022 | Adult ICU | Nurse | S | S | 0 | 0 | 0 |
| 04/14/2022 | Pediatric ICU | Nurse | N | N | S | S | S |
| 04/14/2022 | Pediatric ICU | Nursing Technicians | N | N | S | S | S |
| 04/28/2022 | Adult ICU | Doctor | S | 0 | 0 | 0 | 0 |
| 04/28/2022 | Adult ICU | Doctor | 0 | 0 | S | S | S |
| 04/28/2022 | Adult ICU | Nurse | 0 | 0 | 0 | S | S |
| 04/28/2022 | Pediatric ICU | Nursing Technicians | A | A | 0 | 0 | 0 |
| 04/28/2022 | Neonatal ICU | Doctor | S | S | S | S | S |
| 04/28/2022 | Neonatal ICU | Nurse | S | 0 | 0 | S | 0 |
| 04/28/2022 | Neonatal ICU | Nursing Technicians | S | 0 | 0 | 0 | 0 |
| 05/02/2022 | Adult ICU | Nursing Technicians | N | N | A | A | A |
| 09/19/2022 | Neonatal ICU | Doctor | S | S | 0 | 0 | S |
| 09/23/2022 | Adult ICU | Nurse | N | 0 | 0 | S | S |
| 09/23/2022 | Pediatric ICU | Nurse | N | 0 | N | S | 0 |
| 09/23/2022 | Pediatric ICU | Nurse | S | 0 | 0 | S | S |
| 09/23/2022 | Neonatal ICU | Doctor | S | 0 | 0 | S | S |
| 09/23/2022 | Neonatal ICU | Nurse | 0 | 0 | 0 | S | 0 |
| 09/23/2022 | Adult ICU | Nurse | 0 | 0 | A | 0 | A |
| 09/23/2022 | Adult ICU | Nurse | 0 | 0 | S | 0 | S |
| 10/07/2022 | Adult ICU | Doctor | S | S | S | S | S |
| *Continuation S1 Table:* | | | | | | | |
| 10/07/2022 | Adult ICU | Doctor | S | S | S | S | S |
| 10/07/2022 | Pediatric ICU | Nursing Technicians | N | N | 0 | S | 0 |
| 10/07/2022 | Pediatric ICU | Nursing Technicians | S | A | A | S | S |
| 10/10/2022 | Adult ICU | Nurse | A | 0 | 0 | A | A |
| 10/10/2022 | Adult ICU | Nurse | 0 | 0 | A | S | S |
| 10/24/2022 | Pediatric ICU | Nursing Technicians | S | S | S | S | S |
| 10/24/2022 | Pediatric ICU | Nurse | S | S | 0 | S | S |
| 10/24/2022 | Neonatal ICU | Nurse | A | N | N | S | S |
| 11/03/2022 | Adult ICU | Nurse | A | 0 | N | N | N |
| 11/03/2022 | Adult ICU | Nurse | A | 0 | N | N | N |
| 11/03/2022 | Adult ICU | Nursing student | N | 0 | 0 | 0 | N |
| 11/11/2022 | Pediatric ICU | Nursing Technicians | 0 | 0 | S | S | S |
| 11/11/2022 | Pediatric ICU | Nurse | 0 | 0 | S | S | S |
| 11/11/2022 | Pediatric ICU | Doctor | 0 | 0 | A | A | A |
| 11/11/2022 | Pediatric ICU | Physiotherapist | 0 | 0 | S | S | S |
| 11/14/2022 | Pediatric ICU | Nurse | S | S | A | A | A |
| 11/14/2022 | Pediatric ICU | Nurse | A | A | A | A | A |
| 11/14/2022 | Pediatric ICU | Nurse | N | N | N | S | A |
| 11/14/2022 | Pediatric ICU | Nurse | N | A | S | A | S |
| 11/14/2022 | Pediatric ICU | Nurse | N | N | N | N | N |
| 11/14/2022 | Adult ICU | Nurse | N | N | N | N | N |
| 11/14/2022 | Neonatal ICU | Nurse | S | A | S | S | A |
| 11/16/2022 | Adult ICU | Nurse | N | 0 | 0 | S | S |
| 11/16/2022 | Adult ICU | Nurse | N | N | S | S | S |
| 11/16/2022 | Adult ICU | Doctor | N | S | S | S | S |
| 11/16/2022 | Adult ICU | Nurse | N | 0 | S | S | S |
| 11/16/2022 | Adult ICU | Nurse | N | 0 | S | S | S |
| 11/16/2022 | Adult ICU | Doctor | 0 | 0 | S | S | S |
| 11/21/2022 | Adult ICU | Nurse | N | N | N | N | N |
| 12/01/2022 | Adult ICU | Nurse | 0 | 0 | S | S | S |
| 01/24/2023 | Adult ICU | Doctor | S | S | S | S | S |
| 01/24/2023 | Adult ICU | Nurse | S | S | S | S | S |
| *Continuation S1 Table:* | | | | | | | |
| 01/24/2023 | Adult ICU | Nurse | N | 0 | 0 | A | A |
| 01/30/2023 | Pediatric ICU | Physiotherapist | 0 | 0 | S | S | S |
| 01/30/2023 | Pediatric ICU | Physiotherapist | N | N | S | S | S |
| 01/30/2023 | Pediatric ICU | Nursing Technicians | 0 | 0 | S | S | S |
| 01/30/2023 | Pediatric ICU | Nurse | 0 | 0 | S | S | S |
| 01/31/2023 | Pediatric ICU | Doctor | S | S | S | S | S |
| 01/31/2023 | Pediatric ICU | Nurse | N | 0 | 0 | N | 0 |
| 01/31/2023 | Pediatric ICU | Doctor | S | 0 | 0 | N | N |
| 03/05/2023 | Pediatric ICU | Nurse | N | N | 0 | N | N |
| 03/05/2023 | Adult ICU | Nurse | N | N | N | N | N |
| 03/05/2023 | Pediatric ICU | Nurse | S | S | S | S | S |
| 03/05/2023 | Adult ICU | Nursing Technicians | N | N | N | N | N |
| 03/05/2023 | Adult ICU | Nursing Technicians | N | N | A | A | A |
| 03/05/2023 | Pediatric ICU | Nurse | N | N | A | A | A |
| 03/09/2023 | Pediatric ICU | Nurse | 0 | 0 | S | S | S |
| 04/19/2023 | Pediatric ICU | Nurse | S | S | N | N | N |
| 05/04/2023 | Pediatric ICU | Physiotherapist | 0 | 0 | S | S | S |
| 05/04/2023 | Pediatric ICU | Nurse | N | 0 | S | S | S |
| 05/04/2023 | Pediatric ICU | Nurse | 0 | 0 | S | S | S |
| 07/04/2023 | Adult ICU | Nurse | N | N | 0 | S | 0 |
| 07/04/2023 | Adult ICU | Doctor | N | 0 | 0 | S | 0 |
| 07/04/2023 | Adult ICU | Nurse | N | 0 | 0 | S | 0 |
| 07/04/2023 | Adult ICU | Doctor | N | 0 | 0 | N | 0 |
| 07/04/2023 | Adult ICU | Doctor | N | 0 | 0 | S | 0 |
| 07/04/2023 | Adult ICU | Nursing Technicians | N | 0 | 0 | S | 0 |
| 07/04/2023 | Adult ICU | Nursing Technicians | N | 0 | 0 | S | 0 |
| 07/04/2023 | Adult ICU | Physiotherapist | N | 0 | S | S | 0 |
| 07/20/2023 | Pediatric ICU | Nurse | N | N | 0 | S | S |
| 07/20/2023 | Pediatric ICU | Nurse | N | N | S | S | S |
| 07/20/2023 | Neonatal ICU | Physiotherapist | N | 0 | 0 | N | N |
| 07/20/2023 | Neonatal ICU | Doctor | N | 0 | 0 | N | N |
| 07/20/2023 | Neonatal ICU | Nurse | S | 0 | 0 | S | S |
| *Continuation S1 Table:* | | | | | | | |
| 07/20/2023 | Neonatal ICU | Nurse | A | 0 | 0 | S | S |
| 07/20/2023 | Neonatal ICU | Nurse | N | N | 0 | A | 0 |
| 07/20/2023 | Neonatal ICU | Nursing student | N | N | 0 | N | 0 |
| 07/20/2023 | Neonatal ICU | Physiotherapist | S | 0 | 0 | S | S |
| 07/20/2023 | Neonatal ICU | Nurse | N | 0 | 0 | N | N |
| 07/20/2023 | Neonatal ICU | Nurse | S | S | 0 | S | 0 |
| 07/20/2023 | Neonatal ICU | Nurse | S | S | 0 | S | 0 |
| 07/20/2023 | Neonatal ICU | Nurse | S | 0 | 0 | S | S |
| 07/25/2023 | Adult ICU | Laboratory technician | N | 0 | N | N | N |
| 07/25/2023 | Adult ICU | Nursing Technicians | N | 0 | 0 | A | 0 |
| 07/28/2023 | Adult ICU | Nurse | 0 | 0 | 0 | 0 | N |
| 08/01/2023 | Neonatal ICU | Nurse | N | 0 | 0 | A | 0 |
| 08/01/2023 | Neonatal ICU | Nurse | N | 0 | 0 | S | 0 |
| 08/02/2023 | Adult ICU | Physiotherapist | S | 0 | 0 | S | 0 |
| 08/02/2023 | Pediatric ICU | Doctor | S | 0 | N | S | N |
| 08/02/2023 | Pediatric ICU | Doctor | N | 0 | N | 0 | N |
| 08/02/2023 | Pediatric ICU | Nurse | S | 0 | S | S | S |
| 08/02/2023 | Pediatric ICU | Physiotherapist | N | N | N | N | A |
| 08/02/2023 | Adult ICU | Nurse | S | A | S | S | S |
| 08/02/2023 | Neonatal ICU | Doctor | A | A | A | A | 0 |
| 08/09/2023 | Adult ICU | Nurse | S | A | S | S | S |
| 08/09/2023 | Adult ICU | Nurse | A | N | N | 0 | S |
| 08/10/2023 | Neonatal ICU | Nurse | N | 0 | N | N | 0 |
| 08/10/2023 | Neonatal ICU | Nurse | S | 0 | 0 | S | S |
| 08/10/2023 | Neonatal ICU | Nurse | N | 0 | S | S | S |
| 08/10/2023 | Adult ICU | Nursing student | N | 0 | S | S | S |
| 08/10/2023 | Neonatal ICU | Nurse | S | 0 | 0 | A | A |
| 08/10/2023 | Adult ICU | Nursing Technicians | N | 0 | 0 | S | 0 |
| 08/10/2023 | Adult ICU | Physiotherapist | N | 0 | S | S | 0 |
| 08/10/2023 | Adult ICU | Nursing Technicians | N | 0 | 0 | N | N |
| 08/11/2023 | Neonatal ICU | Nurse | A | A | A | N | N |
| 08/11/2023 | Neonatal ICU | Doctor | N | N | A | A | A |
| *Continuation S1 Table:* | | | | | | | |
| 08/11/2023 | Adult ICU | Nurse | S | A | A | S | S |
| 08/12/2023 | Pediatric ICU | Nurse | A | N | A | A | S |
| 08/14/2023 | Neonatal ICU | Nurse | A | A | A | A | A |
| 08/14/2023 | Neonatal ICU | Nursing student | A | A | A | A | N |
| 08/17/2023 | Adult ICU | Physiotherapist | A | 0 | N | S | 0 |
| 08/17/2023 | Pediatric ICU | Nurse | S | N | N | S | 0 |
| 08/17/2023 | Pediatric ICU | Nurse | N | N | A | S | 0 |
| 08/17/2023 | Pediatric ICU | Physiotherapist | A | A | N | N | N |
| 08/17/2023 | Pediatric ICU | Nurse | N | N | N | N | N |
| 08/23/2023 | Adult ICU | Physiotherapist | A | 0 | A | 0 | S |
| 08/23/2023 | Adult ICU | Physiotherapist | S | A | 0 | S | S |
| 08/25/2023 | Neonatal ICU | Nurse | S | S | A | A | A |
| 08/25/2023 | Neonatal ICU | Nurse | A | A | A | A | A |
| 08/26/2023 | Adult ICU | Nurse | N | 0 | N | N | N |
| 08/26/2023 | Adult ICU | Medical student | A | 0 | A | S | S |
| 08/26/2023 | Adult ICU | Nurse | N | 0 | N | N | N |
| 08/26/2023 | Adult ICU | Nursing Technicians | N | 0 | N | N | N |
| 08/31/2023 | Neonatal ICU | Nurse | S | S | 0 | S | 0 |
| 08/31/2023 | Neonatal ICU | Laboratory technician | S | 0 | 0 | 0 | A |
| 08/31/2023 | Neonatal ICU | Nurse | S | 0 | 0 | S | 0 |
| 08/31/2023 | Neonatal ICU | Nurse | S | 0 | 0 | N | 0 |
| 08/31/2023 | Neonatal ICU | Nurse | S | 0 | S | S | 0 |
| 08/31/2023 | Neonatal ICU | Nurse | S | S | S | S | 0 |
| 08/31/2023 | Neonatal ICU | Physiotherapist | S | 0 | S | S | 0 |
| 08/31/2023 | Neonatal ICU | Physiotherapist | S | 0 | S | S | 0 |
| 08/31/2023 | Neonatal ICU | Nurse | S | 0 | 0 | 0 | S |
| 08/31/2023 | Neonatal ICU | Physiotherapist | S | 0 | S | S | 0 |
| 09/13/2023 | Adult ICU | Nurse | A | A | 0 | A | 0 |
| 09/13/2023 | Adult ICU | Pharmaceutical | A | 0 | N | S | 0 |
| 09/13/2023 | Adult ICU | Doctor | S | 0 | 0 | N | 0 |
| 09/13/2023 | Adult ICU | Pharmaceutical | N | N | N | S | S |
| 10/04/2023 | Adult ICU | Pharmaceutical | S | 0 | A | A | 0 |
| *Continuation S1 Table:* | | | | | | | |
| 10/04/2023 | Adult ICU | Laboratory technician | S | 0 | A | A | A |
| 10/04/2023 | Adult ICU | Nurse | S | 0 | A | 0 | S |
| 10/04/2023 | Adult ICU | Laboratory technician | S | A | A | 0 | S |
| 10/04/2023 | Adult ICU | Nurse | S | A | A | S | 0 |
| 10/11/2023 | Adult ICU | Doctor | S | 0 | A | S | 0 |
| 10/11/2023 | Pediatric ICU | Nurse | S | 0 | N | S | 0 |
| 10/11/2023 | Adult ICU | Doctor | N | 0 | A | S | 0 |
| 10/18/2023 | Adult ICU | Nurse | A | 0 | 0 | A | S |
| 10/18/2023 | Adult ICU | Laboratory technician | S | 0 | A | 0 | 0 |
| 11/18/2023 | Pediatric ICU | Doctor | A | N | 0 | A | N |
| 11/20/2023 | Pediatric ICU | Medical student | N | A | 0 | A | N |
| 11/20/2023 | Pediatric ICU | Medical student | N | A | 0 | A | N |
| 11/20/2023 | Neonatal ICU | Nurse | A | A | S | S | S |
| 12/07/2023 | Adult ICU | Nursing Technicians | N | N | N | N | N |
| 12/07/2023 | Adult ICU | Nurse | S | N | S | S | S |
| 12/07/2023 | Adult ICU | Nurse | S | S | S | S | S |
| 12/07/2023 | Adult ICU | Nursing Technicians | N | N | N | N | N |
| 12/07/2023 | Pediatric ICU | Doctor | N | N | S | S | S |
| 12/13/2023 | Adult ICU | Doctor | N | N | N | N | N |
| 12/13/2023 | Adult ICU | Nurse | N | N | N | N | N |
| 12/13/2023 | Adult ICU | Nurse | N | N | N | N | N |
| 12/13/2023 | Adult ICU | Nurse | N | N | N | N | N |
| 01/10/2024 | Adult ICU | Nurse | S | N | A | S | S |
| 01/10/2024 | Adult ICU | Nurse | S | N | S | S | S |
| 01/10/2024 | Adult ICU | Doctor | S | S | S | S | S |
| 01/10/2024 | Adult ICU | Nurse | N | N | N | S | S |
| 01/12/2024 | Neonatal ICU | Nurse | 0 | 0 | S | S | S |
| 01/12/2024 | Neonatal ICU | Nurse | S | 0 | 0 | S | S |
| 04/12/2024 | Adult ICU | Medical student | N | 0 | 0 | N | 0 |
| 04/22/2024 | Adult ICU | Doctor | N | 0 | 0 | S | 0 |
| 04/22/2024 | Adult ICU | Doctor | N | 0 | 0 | A | 0 |
| 04/22/2024 | Adult ICU | Nursing Technicians | N | 0 | 0 | S | 0 |
| *Continuation S1 Table:* | | | | | | | |
| 04/22/2024 | Adult ICU | Nursing Technicians | N | 0 | 0 | S | 0 |
| 04/23/2024 | Adult ICU | Doctor | S | 0 | 0 | S | 0 |
| 04/23/2024 | Adult ICU | Medical student | S | 0 | 0 | S | 0 |
| 04/30/2024 | Adult ICU | Doctor | A | 0 | 0 | S | 0 |
| 05/28/2024 | Pediatric ICU | Nursing Technicians | N | N | N | S | N |
| 05/28/2024 | Pediatric ICU | Nursing student | N | N | N | A | N |
| 05/30/2024 | Pediatric ICU | Nurse | S | 0 | 0 | S | 0 |
| 05/30/2024 | Adult ICU | Nursing Technicians | S | 0 | 0 | S | 0 |
| 05/30/2024 | Adult ICU | Nursing Technicians | S | 0 | 0 | S | 0 |
| 06/03/2024 | Adult ICU | Nursing Technicians | A | 0 | 0 | A | 0 |
| 06/03/2024 | Adult ICU | Nursing Technicians | N | 0 | 0 | A | 0 |
| 06/19/2024 | Adult ICU | Doctor | N | S | N | N | N |
| 07/01/2024 | Neonatal ICU | Nurse | S | 0 | 0 | S | 0 |
| 07/15/2024 | Adult ICU | Doctor | N | 0 | 0 | N | 0 |
| 07/15/2024 | Pediatric ICU | Doctor | A | 0 | 0 | S | 0 |
| 07/15/2024 | Adult ICU | Doctor | N | 0 | 0 | S | 0 |
| 07/18/2024 | Adult ICU | Nursing Technicians | N | 0 | 0 | S | 0 |
| 07/31/2024 | Adult ICU | Nurse | N | N | N | N | N |
| 07/31/2024 | Adult ICU | Nursing Technicians | N | N | N | S | N |
| 08/08/2024 | Pediatric ICU | Nursing Technicians | A | 0 | N | N | N |
| 08/08/2024 | Neonatal ICU | Nurse | S | S | N | N | N |
| 08/08/2024 | Neonatal ICU | Nurse | S | S | N | S | S |
| 08/12/2024 | Pediatric ICU | Nurse | N | N | N | N | 0 |
| 08/12/2024 | Pediatric ICU | Nurse | N | N | N | N | 0 |
| 08/12/2024 | Pediatric ICU | Physiotherapist | A | 0 | 0 | S | 0 |
| 08/14/2024 | Pediatric ICU | Physiotherapist | A | A | S | S | S |
| 08/14/2024 | Pediatric ICU | Nurse | A | 0 | 0 | S | S |
| 08/19/2024 | Neonatal ICU | Nursing Technicians | A | A | S | S | S |
| 08/19/2024 | Adult ICU | Nurse | A | 0 | 0 | 0 | S |
| 08/19/2024 | Adult ICU | Nurse | N | N | S | S | S |
| 08/19/2024 | Adult ICU | Nurse | N | A | N | N | N |
| 08/19/2024 | Adult ICU | Laboratory technician | N | 0 | 0 | S | S |
| *Continuation S1 Table:* | | | | | | | |
| 08/19/2024 | Pediatric ICU | Nurse | N | 0 | S | S | S |
| 08/19/2024 | Pediatric ICU | Nurse | S | 0 | 0 | A | A |
| 08/19/2024 | Pediatric ICU | Nurse | N | 0 | 0 | S | S |
| 08/19/2024 | Neonatal ICU | Nurse | A | 0 | 0 | S | S |
| 08/19/2024 | Neonatal ICU | Doctor | S | S | S | S | S |
| 08/19/2024 | Pediatric ICU | Doctor | A | A | N | N | N |
| 08/19/2024 | Pediatric ICU | Doctor | A | A | N | N | N |
| 08/19/2024 | Pediatric ICU | Nurse | S | 0 | 0 | S | S |
| 08/20/2024 | Adult ICU | Nurse | A | 0 | N | N | N |
| 08/20/2024 | Adult ICU | Doctor | N | 0 | S | S | S |
| 08/20/2024 | Adult ICU | Doctor | A | A | A | A | A |
| 08/20/2024 | Adult ICU | Nurse | N | 0 | N | N | N |
| 08/22/2024 | Adult ICU | Medical student | S | S | A | S | S |
| 08/28/2024 | Neonatal ICU | Nurse | S | N | 0 | S | N |
| 08/28/2024 | Neonatal ICU | Laboratory technician | N | 0 | 0 | S | N |
| 08/28/2024 | Pediatric ICU | Nurse | N | N | S | S | S |
| 08/28/2024 | Pediatric ICU | Nurse | N | N | 0 | S | S |
| 08/28/2024 | Pediatric ICU | Doctor | N | N | 0 | N | N |
| 08/28/2024 | Pediatric ICU | Nurse | N | N | S | S | 0 |
| 08/28/2024 | Pediatric ICU | Nursing Technicians | N | N | S | S | S |
| 08/29/2024 | Adult ICU | Physiotherapist | A | A | S | S | S |
| 08/29/2024 | Adult ICU | Nursing Technicians | A | A | S | S | S |
| 08/29/2024 | Adult ICU | Nursing Technicians | N | N | S | S | S |
| 09/02/2024 | Adult ICU | Nurse | N | 0 | 0 | 0 | N |
| 09/02/2024 | Adult ICU | Nurse | N | N | N | N | N |
| 09/02/2024 | Adult ICU | Doctor | N | N | S | S | S |
| 09/02/2024 | Adult ICU | Nursing Technicians | N | N | S | S | S |
| 09/05/2024 | Adult ICU | Nursing Technicians | S | S | N | N | N |
| 09/05/2024 | Adult ICU | Nursing Technicians | S | S | S | S | S |
| 09/05/2024 | Adult ICU | Laboratory technician | S | S | A | A | A |
| 09/05/2024 | Pediatric ICU | Nursing Technicians | S | S | S | S | S |
| 09/06/2024 | Neonatal ICU | Nurse | N | 0 | 0 | S | 0 |
| *Continuation S1 Table:* | | | | | | | |
| 09/06/2024 | Neonatal ICU | Nurse | N | 0 | A | N | N |
| 09/07/2024 | Adult ICU | Laboratory technician | N | N | N | N | 0 |
| 09/07/2024 | Adult ICU | Nursing Technicians | N | N | N | N | 0 |
| 09/12/2024 | Adult ICU | Nursing Technicians | A | A | N | N | N |
| 09/12/2024 | Adult ICU | Nursing Technicians | N | 0 | 0 | N | N |
| 09/12/2024 | Pediatric ICU | Doctor | S | S | S | S | S |
| 09/12/2024 | Pediatric ICU | Nurse | S | 0 | S | N | S |
| 09/19/2024 | Adult ICU | Laboratory technician | S | S | S | S | S |
| 09/19/2024 | Adult ICU | Nursing Technicians | N | 0 | 0 | S | S |
| 09/19/2024 | Adult ICU | Nursing Technicians | A | A | S | S | S |
| 09/19/2024 | Pediatric ICU | Doctor | S | S | A | A | A |
| 09/19/2024 | Adult ICU | Doctor | N | N | N | N | N |
| 09/19/2024 | Adult ICU | Nursing Technicians | N | 0 | N | N | N |
| 09/19/2024 | Pediatric ICU | Nursing Technicians | N | N | N | N | N |
| 09/19/2024 | Pediatric ICU | Nursing Technicians | N | N | N | N | N |
| 09/19/2024 | Pediatric ICU | Nursing Technicians | A | A | N | N | N |
| 09/26/2024 | Adult ICU | Nursing Technicians | A | A | A | A | A |
| 09/26/2024 | Neonatal ICU | Nurse | S | S | S | S | S |
| 09/26/2024 | Neonatal ICU | Nurse | S | 0 | 0 | S | S |
| 09/26/2024 | Neonatal ICU | Nurse | S | S | 0 | S | S |
| 09/26/2024 | Neonatal ICU | Nurse | S | S | 0 | S | S |
| 09/26/2024 | Neonatal ICU | Nurse | S | S | S | S | S |
| 09/26/2024 | Adult ICU | Nurse | N | N | N | N | S |
| 09/26/2024 | Neonatal ICU | Laboratory technician | S | 0 | 0 | N | N |
| 09/26/2024 | Neonatal ICU | Laboratory technician | S | 0 | 0 | 0 | N |
| 09/26/2024 | Adult ICU | Medical student | N | N | N | N | N |
| 09/26/2024 | Neonatal ICU | Nurse | S | S | S | S | S |
| 10/03/2024 | Adult ICU | Nurse | N | 0 | 0 | A | N |
| 10/03/2024 | Adult ICU | Nurse | N | N | N | S | N |
| 10/04/2024 | Adult ICU | Nursing Technicians | A | A | S | S | S |
| 10/04/2024 | Adult ICU | Nursing Technicians | N | 0 | N | S | S |
| 10/10/2024 | Adult ICU | Medical student | A | A | A | A | A |
| *Continuation S1 Table:* | | | | | | | |
| 10/10/2024 | Adult ICU | Medical student | A | 0 | 0 | A | A |
| 10/10/2024 | Adult ICU | Doctor | A | A | A | A | A |
| 10/10/2024 | Adult ICU | Doctor | A | A | A | A | A |
| 10/17/2024 | Adult ICU | Nurse | S | S | A | A | A |
| 10/17/2024 | Adult ICU | Nurse | A | A | A | A | A |
| 10/17/2024 | Adult ICU | Nurse | A | 0 | A | A | A |
| 10/24/2024 | Adult ICU | Nursing Technicians | A | A | S | S | S |
| 10/28/2024 | Adult ICU | Nurse | S | 0 | S | S | 0 |
| 10/28/2024 | Adult ICU | Nurse | N | 0 | 0 | S | 0 |
| 10/28/2024 | Adult ICU | Nurse | S | 0 | 0 | S | 0 |
| 10/28/2024 | Adult ICU | Doctor | S | S | 0 | S | 0 |
| 10/28/2024 | Adult ICU | Nurse | S | 0 | 0 | S | 0 |
| 10/28/2024 | Adult ICU | Doctor | S | 0 | 0 | S | 0 |
| 11/07/2024 | Adult ICU | Nurse | A | 0 | A | A | 0 |
| 11/07/2024 | Adult ICU | Nurse | A | 0 | A | 0 | 0 |
| 12/18/2024 | Neonatal ICU | Nurse | A | A | A | A | A |
| 12/27/2024 | Adult ICU | Nurse | N | 0 | 0 | S | 0 |
| 12/27/2024 | Adult ICU | Nurse | N | N | N | S | 0 |
| 12/27/2024 | Adult ICU | Laboratory technician | N | N | N | N | 0 |

**N: not hand hygiene compliance; A: alcohol; S: soap; 0: not Applicable.**
