# Supplementary material for: Assessing the COVID-19 legacy on hand hygiene: Retrospective observational before–after study of compliance and alcohol-based
Source: PLOS Glob Public Health. 2026 Feb 27;6(2):e0005210. doi: 10.1371/journal.pgph.0005210 (PMC12948101; doi:10.1371/journal.pgph.0005210)
Supplement: S2 Table — Aggregated data covering the period from January 9, 2017, to January 8, 2018, in adult, pediatric, and neonatal intensive care units. (DOCX) [file pgph.0005210.s002.docx]

**Supplementary DataSet**

**S2 Table.** Opportunities for Hand Hygiene Before the COVID-19 Pandemic. Aggregated data covering the period from January 9, 2017, to January 8, 2018.

| **Adult, Pediatric, and Neonatal Intensive Care Units** | |
| --- | --- |
| Opportunities | 1048 |
| HH compliance | 640 |
