# Supplementary material for: Assessing the COVID-19 legacy on hand hygiene: Retrospective observational before–after study of compliance and alcohol-based
Source: PLOS Glob Public Health. 2026 Feb 27;6(2):e0005210. doi: 10.1371/journal.pgph.0005210 (PMC12948101; doi:10.1371/journal.pgph.0005210)
Supplement: S3 Table — Compliance rates calculated as hand hygiene actions divided by observed opportunities. (DOCX) [file pgph.0005210.s003.docx]

**Supplementary DataSet**

**S3 Table.** Rate of Hand Hygiene Compliance (HH actions/opportunities) in Adult, Pediatric, and Neonatal ICUs Before the COVID-19 Pandemic.

| **Sector** | **HH compliance rate** |
| --- | --- |
| Overall HH | 61% (640/1048) |
| Adult ICU | 49,67% |
| Pediatric ICU | 67,51% |
| Neonatal ICU | 64,78% |
