# Supplementary material for: Assessing the COVID-19 legacy on hand hygiene: Retrospective observational before–after study of compliance and alcohol-based
Source: PLOS Glob Public Health. 2026 Feb 27;6(2):e0005210. doi: 10.1371/journal.pgph.0005210 (PMC12948101; doi:10.1371/journal.pgph.0005210)
Supplement: S4 Table — Compliance rates stratified by professional category. (DOCX) [file pgph.0005210.s004.docx]

**Supplementary DataSet**

**S4 Table.** Rate of Hand Hygiene Compliance (HH actions/opportunities) by Occupational Category Before the COVID-19 Pandemic.

| **Professional Category** | **Rate of HHC (HH actions/opportunities)** |
| --- | --- |
| Doctors | 70,8% (114/161) |
| Students | 67,12% (49/73) |
| Nurses | 62,78% (194/309) |
| Nursing Technicians | 54,18% (207/382) |
| Other Professionals | 61,78% (76/123) |
