# Supplementary material for: Assessing the COVID-19 legacy on hand hygiene: Retrospective observational before–after study of compliance and alcohol-based
Source: PLOS Glob Public Health. 2026 Feb 27;6(2):e0005210. doi: 10.1371/journal.pgph.0005210 (PMC12948101; doi:10.1371/journal.pgph.0005210)
Supplement: S5 Table — Distribution of hand hygiene actions according to product used (liquid soap or alcohol-based preparation) and mean hand rubbing time. (DOCX) [file pgph.0005210.s005.docx]

**Supplementary DataSet**

**S5 Table.** Hand Hygiene (HH) Compliance rate by HH product before the COVID-19 pandemic.

| **Preparation** | **Rafe of HHC (%)** |
| --- | --- |
| Liquid Soap | 87% |
| Alcohol-based preparations | 13% |
|  |  |
| *Hand rubbing time* | *10,3 +- 4,08 seg* |
