# Supplementary material for: Assessing the COVID-19 legacy on hand hygiene: Retrospective observational before–after study of compliance and alcohol-based
Source: PLOS Glob Public Health. 2026 Feb 27;6(2):e0005210. doi: 10.1371/journal.pgph.0005210 (PMC12948101; doi:10.1371/journal.pgph.0005210)
Supplement: S6 Table — Aggregated quarterly data summarizing total opportunities, number of hand hygiene compliance, and type of product used. (DOCX) [file pgph.0005210.s006.docx]

**Supplementary DataSet**

**S6 Table.** Analysis of Hand Hygiene Opportunities, Compliance, and Use of Alcohol- or Soap-Based Formulations During the COVID-19 Pandemic.

|  | **Opportunities** | **Hand Hygiene Compliance** | **Alcohol** | **Soap** |
| --- | --- | --- | --- | --- |
| Oct–Dec 2021 | 458 | 297 | 89 | 208 |
| Jan–Mar 2022 | 140 | 109 | 74 | 31 |
| Apr–Jun 2022 | 82 | 72 | 6 | 66 |
| Jul–Sep 2022 | 20 | 17 | 2 | 15 |
| Oct–Dec 2022 | 127 | 90 | 25 | 65 |
| Jan–Mar 2023 | 69 | 44 | 8 | 36 |
| Apr–Jun 2023 | 15 | 11 | 0 | 11 |
| Jul–Sep 2023 | 256 | 162 | 56 | 106 |
| Oct–Dec 2023 | 94 | 53 | 23 | 30 |
| Jan–Mar 2024 | 26 | 21 | 1 | 20 |
| Apr–Jun 2024 | 41 | 22 | 6 | 16 |
| Jul–Sep 2024 | 325 | 188 | 46 | 142 |
| Oct–Dec 2024 | 88 | 71 | 45 | 26 |
