# Supplementary material for: Assessing the COVID-19 legacy on hand hygiene: Retrospective observational before–after study of compliance and alcohol-based
Source: PLOS Glob Public Health. 2026 Feb 27;6(2):e0005210. doi: 10.1371/journal.pgph.0005210 (PMC12948101; doi:10.1371/journal.pgph.0005210)
Supplement: S7 Table — Quarterly compliance data including moving average and trend estimates. (DOCX) [file pgph.0005210.s007.docx]

**Supplementary DataSet**

**S7 Table.** Time-Series Analysis of Hand Hygiene Compliance, Moving Average and e Trend during the COVID-19 Pandemic.

|  | **Hand Hygiene Compliance** | **Moving Average (quarterly)** | **Trend (%)** |
| --- | --- | --- | --- |
| Oct–Dec 2021 | 297 |  |  |
| Jan–Mar 2022 | 109 |  | -0,632996633 |
| Apr–Jun 2022 | 72 | 159,3333333 | -0,339449541 |
| Jul–Sep 2022 | 17 | 66 | -0,763888889 |
| Oct–Dec 2022 | 90 | 59,66666667 | 4,294117647 |
| Jan–Mar 2023 | 44 | 50,33333333 | -0,511111111 |
| Apr–Jun 2023 | 11 | 48,33333333 | -0,75 |
| Jul–Sep 2023 | 162 | 72,33333333 | 13,72727273 |
| Oct–Dec 2023 | 53 | 75,33333333 | -0,672839506 |
| Jan–Mar 2024 | 21 | 78,66666667 | -0,603773585 |
| Apr–Jun 2024 | 22 | 32 | 0,047619048 |
| Jul–Sep 2024 | 188 | 77 | 7,545454545 |
| Oct–Dec 2024 | 71 | 93,66666667 | 0,002008828 |
