# Supplementary material for: Assessing the COVID-19 legacy on hand hygiene: Retrospective observational before–after study of compliance and alcohol-based
Source: PLOS Glob Public Health. 2026 Feb 27;6(2):e0005210. doi: 10.1371/journal.pgph.0005210 (PMC12948101; doi:10.1371/journal.pgph.0005210)
Supplement: S8 Table — Quarterly number of observed opportunities with corresponding moving averages and trend estimates. (DOCX) [file pgph.0005210.s008.docx]

**Supplementary DataSet**

**S8 Table.** Time-Series Analysis of Opportunities, Moving Average and e Trend during the COVID-19 Pandemic.

|  | **Opportunities** | **Moving Average (quarterly)** | **Trend (%)** |
| --- | --- | --- | --- |
| Oct–Dec 2021 | 458 |  |  |
| Jan–Mar 2022 | 140 |  | -0,694323144 |
| Apr–Jun 2022 | 82 | 226,6666667 | -0,414285714 |
| Jul–Sep 2022 | 20 | 80,66666667 | -0,756097561 |
| Oct–Dec 2022 | 127 | 76,33333333 | 5,35 |
| Jan–Mar 2023 | 69 | 72 | -0,456692913 |
| Apr–Jun 2023 | 15 | 70,33333333 | -0,782608696 |
| Jul–Sep 2023 | 256 | 113,3333333 | 16,06666667 |
| Oct–Dec 2023 | 94 | 121,6666667 | -0,6328125 |
| Jan–Mar 2024 | 26 | 125,3333333 | -0,723404255 |
| Apr–Jun 2024 | 41 | 53,66666667 | 0,576923077 |
| Jul–Sep 2024 | 325 | 130,6666667 | 6,926829268 |
| Oct–Dec 2024 | 88 | 151,3333333 | 0,000833136 |
