# Supplementary material for: Assessing the COVID-19 legacy on hand hygiene: Retrospective observational before–after study of compliance and alcohol-based
Source: PLOS Glob Public Health. 2026 Feb 27;6(2):e0005210. doi: 10.1371/journal.pgph.0005210 (PMC12948101; doi:10.1371/journal.pgph.0005210)
Supplement: S9 Table — Quarterly compliance rates expressed as percentages, including moving averages and trend estimates. (DOCX) [file pgph.0005210.s009.docx]

**Supplementary DataSet**

**S9 Table.** Time-Series Analysis of Hand Hygiene Compliance (HHC)/Opportunities, Moving Average and e Trend during the COVID-19 Pandemic.

|  | **HHC/Opportunities (%)** | **Moving Average (quarterly)** | **Trend (%)** |
| --- | --- | --- | --- |
| Oct–Dec 2021 | 64,85% |  |  |
| Jan–Mar 2022 | 77,86% |  | 20,06% |
| Apr–Jun 2022 | 87,80% | 76,84% | 12,78% |
| Jul–Sep 2022 | 85,00% | 83,55% | -3,19% |
| Oct–Dec 2022 | 70,87% | 0,812236733 | -16,63% |
| Jan–Mar 2023 | 63,77% | 0,732114192 | -10,02% |
| Apr–Jun 2023 | 73,33% | 0,693225303 | 15,00% |
| Jul–Sep 2023 | 63,28% | 0,667942331 | -0,137073864 |
| Oct–Dec 2023 | 56,38% | 0,643325207 | -0,109009719 |
| Jan–Mar 2024 | 80,77% | 0,668111532 | 0,432510885 |
| Apr–Jun 2024 | 53,66% | 0,63603582 | -0,335656214 |
| Jul–Sep 2024 | 57,85% | 0,640913071 | 7,80% |
| Oct–Dec 2024 | 80,68% | 0,640621695 | 241,12% |
