# Supplementary material for: Assessing the COVID-19 legacy on hand hygiene: Retrospective observational before–after study of compliance and alcohol-based
Source: PLOS Glob Public Health. 2026 Feb 27;6(2):e0005210. doi: 10.1371/journal.pgph.0005210 (PMC12948101; doi:10.1371/journal.pgph.0005210)
Supplement: S2 Fig — (DOCX) [file pgph.0005210.s011.docx]

**Supplementary DataSet**

**S2 Fig. Time-Series Analysis of Opportunities, Moving Average and e Trend during the COVID-19 Pandemic.**
