# Supplementary material for: Assessing the COVID-19 legacy on hand hygiene: Retrospective observational before–after study of compliance and alcohol-based
Source: PLOS Glob Public Health. 2026 Feb 27;6(2):e0005210. doi: 10.1371/journal.pgph.0005210 (PMC12948101; doi:10.1371/journal.pgph.0005210)
Supplement: Attachment — Submitted filename: Response to Reviewers.docx [file pgph.0005210.s014.docx]

**RESPONSE TO REVIEWERS**

| **Reviewer Comments** | **Response to Reviewers** |
| --- | --- |
| Reviewer #1: The study design is not mentioned in the manuscript and further enough details about the study are not provided in the methods section to understand and interpret results in a meaningful manner. Based on the information provided study design does not seem appropriate to answer the research question. | We agree. We have now clearly described the study as a retrospective observational before–after study in both the Abstract and the first paragraph of the Methods section.  We agree that the previous version of the manuscript did not provide sufficient methodological detail, which may have created the impression that the study design was inadequate for the research question.  Our intention was not to establish causal relationships, but rather to describe changes in hand hygiene compliance and product use across two distinct surveillance periods (pre- and post-pandemic), using routinely collected institutional audit data. For this descriptive and comparative aim, a retrospective observational before–after design is methodologically appropriate and is consistent with prior literature evaluating changes in hand hygiene behavior.  At the end of the Introduction, we added a clarifying sentence explicitly stating that the study is descriptive rather than causal. The new sentence reads: *“Rather than establishing causal relationships, our objective was to characterize patterns of compliance and product use across two distinct time periods shaped by different operational and infection-control conditions.”* |
| **Reviewer #1 Comment 2:**  “Further enough details about the study are not provided in the methods section.” | We agree that the previous version of the Methods section did not provide sufficient detail regarding the study design and operational procedures, which may have limited the reader’s ability to interpret the findings.  To address this, we **substantially revised and expanded the entire Methods section** to clearly describe the design, rationale, procedures, and analytical approach of the study. The following key modifications were made:   1. **Explicit identification of the study design** as a *retrospective observational before–after study*, including a brief justification explaining why this design is appropriate for a descriptive assessment of hand hygiene patterns rather than for causal inference. 2. **Expanded description of the study setting**, specifying the type of hospital and the three intensive care units included. 3. **Clear definition of participants and units of observation**, emphasizing that the unit of analysis was the hand hygiene opportunity, with explicit alignment to WHO definitions. 4. **Detailed explanation of the time windows**, including dates, rationale for the different durations, and implications for interpretation. 5. **Comprehensive clarification of observation procedures**, including who performed observations, training and supervision, covert data collection, session duration, distribution across shifts and days, and standardized criteria for classifying hand hygiene opportunities. 6. **Precise definition of a hand hygiene opportunity**, noting that each WHO moment was counted separately in the post-pandemic period. 7. **Clarification of sampling strategy**, stating that all available surveillance data were included and explaining why no a priori sample size calculation was applicable. 8. **Description of bias-control measures**, including mitigation of the Hawthorne effect, observer training, and acknowledgement of the absence of interobserver agreement statistics. 9. **Revised statistical analysis section**, now concise, aligned with the analyses reported in the Results, and focused on the tests appropriate for comparing proportions and categorical variables.   These changes make the study design explicit, strengthen the methodological transparency, and ensure that the design is clearly aligned with the descriptive nature of the research question.  **Changes in manuscript:** Revisions made to the Methods section throughout (Study Design; Setting; Participants and Units of Observation; Time Windows; Observation Procedures; Definition of HH Opportunity; Sampling Strategy; Bias Control; Statistical Analysis). |
| Reviewer #2: Title: Consider adding study design Abstract: Can include exact time windows (pre: 09/01/2017–08/01/2018; post: 10/29/2021–12/27/2024) for better clarity. Introduction: Can add a brief mention about the importnace of product choice (soap vs alcohol) adds value beyond compliance percentages. Methods: 1. To mention the study design clearly. 2. The manuscript lacks clarity regarding who were the observers infection-control staff, external auditors, peers? Were observations overt or covert? This is crucial because overt observation inflates compliance via the Hawthorne effect. Add explicit text describing observers, training, blinding, inter-rater reliability. 3. The denominator definition (HM opportunities) must be precise. Did each WHO moment count separately? 4. Was there any sampling techniques used for sampling the oppurtunities? Justify. 5. Explain about the observation sessions, how many per week, duration per session, days of week, shift times? Large post-pandemic period (Oct 2021–Dec 2024) vs pre-pandemic one year (Sep 2017–Aug 2018): explain why windows differ in length and how that could bias findings. 5. No inter-observer agreement statistics are reported. If multiple observers collected data, report kappa or percent agreement; if not available, state this as a limitation and describe training. Over a multi-year post-period, observer behaviour may change; mention possible inter-observer variability. 6. There was no mention about the sample size calculation. Please explain. 7. Confounding by other interventions-During 2019–2024 there could be multiple simultaneous interventions (education campaigns, supply chain improvements, local policy changes). Authors should list local HH interventions and, if data exist, adjust or stratify analyses. Results: Time series plot (if dates available) showing monthly/quarterly compliance to visualise trend and decay can be done. If data permits interrupted time series analysis can be considered. Discussion: 1. To discuss about the observer bias and how it might have affected the study results. 2. Because only one hospital is studied, external validity is limited — mention this explicitly. 3. Discuss and mention in limitation that causality cannot be inferred due to the observational before–after design and potential confounding.  Whole manuscript: Proofread for grammar and consistent tense. Ensure all acronyms defined. | We thank Reviewer #2 for the thorough and constructive feedback. We have carefully revised the manuscript to address all points raised. Below, we provide a detailed response to each comment and indicate where changes were made.  **Title**  **Reviewer comment:** *“Consider adding study design.”* **Response:** We agree and have revised the title to explicitly include the study design. **Changes in manuscript:** Title updated to include “retrospective observational before–after study.”  **Abstract**  **Reviewer comment:** *“Include exact time windows (pre and post) for clarity.”* **Response:** The requested time windows were added to improve clarity. **Changes:** Abstract now includes “pre-pandemic (09/01/2017–08/01/2018) and post-pandemic (10/29/2021–12/27/2024).”  **Introduction**  **Reviewer comment:** *“Add mention of the importance of product choice (soap vs alcohol).”* **Response:** A concise paragraph was added emphasizing why product choice provides insight beyond compliance percentages, using recent literature to support this point. **Changes:** New sentences added in the final part of the Introduction referencing the role of product choice (Lotfinejad et al., Verwilghen et al., Vermeil et al., Glowicz et al.).  **METHODS — Major Revisions (Items 1–7)**  **1. Study design clearly stated**  **Reviewer comment:** *“To mention the study design clearly.”* **Response:** The study design is now explicitly described as a *retrospective observational before–after study*, with justification for its use. **Changes:** Section “Study design and setting” revised accordingly.  **2. Observers, training, overt/covert, inter-rater reliability**  **Reviewer comment:** *“Clarify observers, training, blinding, overt vs covert, inter-rater reliability.”* **Response:** We fully revised the observation procedures. We clarified that:  observations were **covert** (to minimize Hawthorne effect),  observers were **medical students** trained with WHO materials,  supervision was performed by **infection-control nurses**,  interobserver agreement statistics were not recorded historically, which is now explicitly acknowledged as a limitation. **Changes:** Section “Observation procedures” completely rewritten.  **3. Denominator definition / WHO moments**  **Reviewer comment:** *“Denominator must be precise. Did each WHO moment count separately?”* **Response:** Yes. Each WHO moment was counted separately during the post-pandemic period, and this is now clearly stated. **Changes:** Section “Definition of hand hygiene opportunity” revised.  **4. Sampling technique**  **Reviewer comment:** *“Was any sampling used? Justify.”* **Response:** No sampling was used; all available opportunities were included. We added an explanation of why sampling was not applicable given the surveillance-based nature of the data. **Changes:** Section “Sampling strategy and sample size” added.  **5. Observation sessions; session frequency; shifts; unequal windows**  **Reviewer comment:** *“Explain observation sessions, shifts, and differences in time windows.”* **Response:** We added detailed descriptions of:  session duration (≈30 minutes),  distribution across morning/afternoon/night shifts and weekdays,  coverage of all ICUs,  rationale for the different time windows (change in institutional auditing post-pandemic). **Changes:** Sections “Time windows and rationale” and “Observation procedures” revised.  **6. Interobserver variability**  **Reviewer comment:** *“Report kappa if available or acknowledge limitation.”* **Response:** Interobserver agreement statistics were not recorded in the hospital’s surveillance system. This is now explicitly acknowledged as a study limitation. **Changes:** Added to both “Observation procedures” and “Limitations.”  **7. Sample size calculation**  **Reviewer comment:** *“No mention of sample size calculation.”* **Response:** We clarified that, because the study relies on routine surveillance data, no a priori sample size calculation was applicable. **Changes:** Included in “Sampling strategy and sample size.”  **8. Confounding by interventions (2019–2024)**  **Reviewer comment:** *“List local HH interventions and discuss confounding.”* **Response:** We explained that the hospital conducts semiannual hand hygiene educational campaigns but that no major intervention occurred specifically within our time windows. Nonetheless, we acknowledge possible residual confounding from unrecorded or local activities. **Changes:** Added to “Bias control” and reinforced in “Limitations.”  **RESULTS**  **Time-series plot / ITS recommendation**  **Reviewer comment:** *“Consider adding a time series plot; ITS if possible.”* **Response:** A quarterly time-series plot of post-pandemic compliance and product use has now been added. ITS was not feasible due to lack of timestamp granularity in pre-pandemic data. **Changes:** Figure added; explanation inserted in “Statistical analysis” and Results.  **DISCUSSION**  **1. Observer bias**  **Reviewer comment:** *“Discuss observer bias.”* **Response:** A paragraph discussing observer bias, Hawthorne effect mitigation, and interobserver variability has been added. **Changes:** Added to Discussion (final section).  **2. External validity**  **Reviewer comment:** *“Only one hospital; mention external validity limitation.”* **Response:** We added a statement acknowledging limited generalizability due to the single-center design. **Changes:** Added to Discussion (Limitations).  **3. Causal inference**  **Reviewer comment:** *“Mention that causality cannot be inferred.”* **Response:** A sentence explicitly stating that causal inferences cannot be made given the retrospective before–after design has been added. **Changes:** Included in Discussion (Limitations).  **MANUSCRIPT-WIDE EDITING**  **Grammar, tenses, acronym consistency**  **Reviewer comment:** *“Proofread; ensure acronyms defined.”* **Response:** The manuscript was carefully proofread for grammar and consistency. All acronyms (HH, ICU, ABHR, WHO) are now defined at first appearance. **Changes:** Throughout the manuscript. |

We thank the reviewer for the insightful and detailed comments, which substantially improved the clarity, methodological transparency, and interpretability of our manuscript. All suggestions were addressed, and the revised version incorporates the requested modifications throughout the title, abstract, introduction, methods, results, discussion, and limitations.
