# Supplementary material for: Assessing the COVID-19 legacy on hand hygiene: Retrospective observational before–after study of compliance and alcohol-based
Source: PLOS Glob Public Health. 2026 Feb 27;6(2):e0005210. doi: 10.1371/journal.pgph.0005210 (PMC12948101; doi:10.1371/journal.pgph.0005210)
Supplement: Attachment — Submitted filename: Response_to_Reviewers_auresp_2.docx [file pgph.0005210.s015.docx]

RESPONSE TO REVIEWERS

We sincerely thank the Academic Editor for the careful re-evaluation of our manuscript and for the constructive feedback provided. We greatly appreciate the recognition that the topic is well researched and the guidance to further clarify the manuscript’s contribution to existing knowledge.

In response, we undertook a focused and structured revision aimed at (i) making the study’s conceptual gap more explicit, (ii) improving conciseness by removing redundancy, and (iii) ensuring alignment between the Introduction, Summary, and Discussion. These revisions were motivated by the need to clearly articulate why this study is necessary within an already well-established field and how it extends current evidence.

| Reviewer Comments | Response to Reviewers |
| --- | --- |
| Reviewer #1 Comment 1: This is a well researched topic. Therefore, please mention what this article adds to the existing knowledge. | We agree with the Editor that hand hygiene is a well-studied topic and appreciate the opportunity to clarify the specific contribution of our work. To address this comment, we revised both the Introduction and the Summary to explicitly state the conceptual gap that motivated the study and to clearly position our findings within the existing literature.  While increased hand hygiene compliance during the acute phase of the COVID-19 pandemic is well documented, we identified a gap in empirical evidence regarding the sustainability and contextual variability of these behaviors once emergency conditions subside and routine clinical practice resumes. In particular, limited data are available on how compliance patterns, adherence to specific WHO hand hygiene moments, and product choice evolve over extended post-pandemic periods and across different intensive care contexts.  To make this gap explicit, we restructured the Introduction following a clear logical progression: (i) summarizing established evidence that crises increase hand hygiene adherence; (ii) highlighting that these improvements are often temporary and not automatically sustained; (iii) identifying the lack of long-term, context-sensitive post-pandemic data; and (iv) clearly stating how our study addresses this gap by evaluating sustainability, heterogeneity across adult, pediatric, and neonatal ICUs, and changes in hand hygiene behavior beyond binary compliance measures.  Accordingly, the final paragraph of the Introduction now explicitly states that our study examines long-term post-pandemic sustainability using real-world surveillance data, focusing on variability across ICU settings, WHO hand hygiene moments, and product choice. The Summary was also revised to reflect this framing, ensuring that readers can readily identify the study’s contribution even when reading the abstract alone. |
| Reviewer #1 Comment 2: There is a duplication of many information in the manuscript, such as the "my five moments of HH", which comes in the introduction as well as in the methods section. Paragraph from lines 77-85 can be removed from Introduction as it fits more in the discussion. | We thank the reviewer for this valuable observation and fully agree that the manuscript contained unnecessary duplication regarding the description of the WHO “My Five Moments for Hand Hygiene.”  In response to this comment, we revised the manuscript to eliminate redundancy and improve narrative clarity. The explanatory paragraph previously located in the Introduction (lines 77–85) was removed, as suggested.  In the Introduction, references to the WHO framework were streamlined to a brief contextual mention without detailed explanation, while interpretative discussion related to differences across hand hygiene moments was retained exclusively in the Discussion section. These changes reduce repetition, improve conciseness, and strengthen the logical separation between background, methodology, and interpretation.  This revision contributes to shortening the manuscript and aligning its structure with journal standards, as recommended by the Editor. |
| Reviewer #1 Comment 3: The methods section has paragraphs which should be in discussion, such as the limitations. Paragraph from lines 215-223 should also be in the discussion section. | We thank the reviewer for this important comment and agree that the placement of methodological limitations within the Methods section was not optimal.  In response, we revised the manuscript to improve structural clarity and adherence to the IMRAD format. The paragraph describing study limitations (previously located in the Methods section, lines 215–223) was removed from the Methods and relocated to the Discussion section, where it is now presented in an interpretative context alongside the study findings.  This revision ensures a clearer distinction between methodological description and interpretative discussion, reduces redundancy, and improves the overall readability and organization of the manuscript. |
| Reviewer #1 Comment 4: The reasons for lower volume of observations during the April-June 2023 should be discussed. | We thank the reviewer for this pertinent comment and agree that the marked reduction in the number of observations during April–June 2023 required explicit clarification.  In response, we revised the Discussion to address this issue directly. We now explain that the lower volume of hand hygiene observations during this period reflects a temporary reduction in observation capacity rather than a true decrease in hand hygiene activity or compliance. This reduction was primarily attributable to operational and logistical factors related to disruptions in the academic calendar following the COVID-19 pandemic, which affected the availability of trained observers, including medical students involved in routine surveillance activities.  We further clarify that this decrease was isolated and time-limited, as evidenced by the subsequent recovery of observation volume in later quarters. Importantly, this fluctuation did not coincide with a sustained decline in hand hygiene compliance, supporting the interpretation that the observed reduction represents a surveillance artifact rather than a behavioral change among healthcare workers.  These clarifications were added to the Discussion section to ensure transparency in data interpretation and to distinguish variations in observation intensity from true changes in hand hygiene practices. |
| Reviewer #1 Comment 5: Discussion can be made more succinct. | We thank the reviewer for this important comment and agree that the Discussion could be further streamlined. In response, we undertook a focused revision to improve conciseness while preserving the scientific content and interpretative depth.  Specifically, we reduced redundancy by removing repeated conceptual descriptions already addressed in the Introduction, particularly regarding WHO hand hygiene initiatives and the “Five Moments” framework. The Discussion was refocused on interpretation rather than background, with greater emphasis on how our findings address the identified gap in post-pandemic sustainability of hand hygiene practices.  We also condensed sections discussing alcohol-based hand rub use by integrating evidence on usability, institutional culture, and international recommendations into a single, more cohesive paragraph. In addition, temporal variability and the temporary reduction in observation volume during early 2023 were consolidated into a unified interpretative section to avoid repetition and improve narrative flow.  As a result of these changes, the Discussion is substantially shorter and more focused, explicitly highlighting the study’s contribution: demonstrating that post-pandemic hand hygiene sustainability is partial, context-dependent, and accompanied by meaningful behavioral transformation rather than uniform persistence. We believe these revisions have improved clarity, readability, and alignment with the journal’s expectations. |

Once again, we thank the reviewer for the valuable comments, which substantially improved the organization and readability of the manuscript.
